# Behind the Mask: Detection and Characterization of Benzalkonium Chloride Adulteration in Immunoassay-Based Urine Drug Testing for Tetrahydrocannabinol (THC)

**DOI:** 10.1021/acsomega.6c03171

**Published:** 2026-06-19

**Authors:** Éabha Finn, Jennifer Huffman, Kristen J. Skogerboe

**Affiliations:** Department of Chemistry, 7283 Seattle University, Seattle, Washington 98122, United States

## Abstract

Urine drug testing for tetrahydrocannabinol (THC) is widely performed using lateral flow immunoassay (LFI) kits. However, these tests are vulnerable to adulteration by benzalkonium chloride (BAK), a cationic surfactant that is a common ingredient in consumer products, such as eyedrops and antiseptics, which can produce false-negative THC results. BAK is believed to act by forming micelles around the THC metabolite 11-nor-9-carboxy-tetrahydrocannabinol (THC–COOH), blocking detection. Because BAK is not routinely included in specimen validity testing panels, it remains a significant potential masking agent in urine drug screening. This study evaluated BAK-containing products and confirmed that readily available commercial formulations can effectively mask THC in the urine. For example, when 25 mL of an antiseptic liquid containing BAK at a nominal concentration of 1300 ppm was added to 75 mL of urine, the LFI test was found to flip from true positive to false negative. Consumer products containing lower concentrations of BAK, such as eye drops, typically mask THC detection when added to urine at an equal volume. To address the vulnerability of LFI-based THC testing to BAK addition, a rapid, low-cost, noninstrumental screening approach based on the standard addition method was developed to distinguish true negatives from BAK-induced false negatives. In blinded testing of 45 urine samples, consisting of 15 true positives, 15 true negatives, and 15 false negatives, the method correctly identified all of the adulterated samples. These findings highlight a practical strategy for improving the reliability of THC urine drug screening in settings where LFI kits are routinely used.

## Graphical abstract

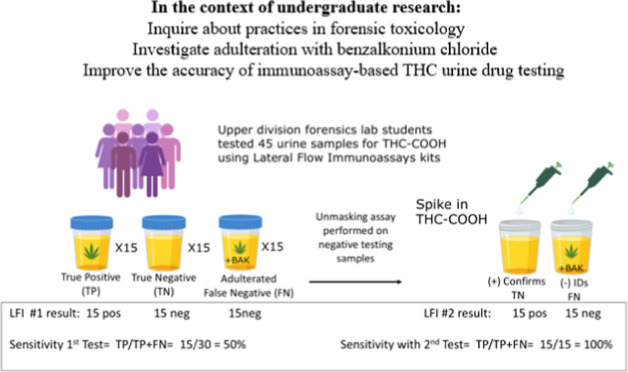

## 1 Introduction

Urine drug testing is used widely in clinical, workplace, athletic, legal, and at-home settings to monitor recent substance use. According to the 2023 National Survey on Drug Use and Health (NSDUH), 70.5 million (24.9%) individuals aged 12 or older reported illicit drug use in the previous year, including 61.8 million marijuana users.^1^ The global drug-testing market reflects this high prevalence and is projected to exceed $6 billion USD by 2025.^2^

Urine is a preferred matrix for drug testing because it is easily collected, generally contains detectable analyte concentrations, is simple to process, and has well-characterized, extended detection windows that define the period after use during which prior exposure to a substance can be identified.^3^ However, unobserved collection can be less reliable than blood, saliva, or hair sampling because donor manipulation cannot be ruled out. Known modes of urine sample tampering include dilution, substitution, and adulteration, all of which can compromise test sensitivity.^4,5^ In this case, test sensitivity is the ability to correctly identify people who have tampered with their urine sample, thus impairing the overall accuracy of the test. Dilution is typically achieved by adding water to reduce drug concentrations below the threshold for a positive result. Substitution often involves submitting an external, drug-free urine sample in place of the donor’s specimen. Adulteration is the intentional addition of substances other than water to a urine specimen to mask a positive result and produce a false negative.

Accuracy in urine drug testing is essential, because results can have important medical, legal, and employment consequences. Two key measures of the test performance are sensitivity and specificity. Sensitivity is the ability to correctly identify true positives and is calculated as true positives divided by the sum of true positives and false negatives. Specificity reflects the ability to correctly identify true negatives and is calculated as true negatives divided by the sum of true negatives and false positives. Together, these metrics describe the overall test reliability. Urine samples that contain drugs but have been diluted or adulterated may yield false-negative results, reducing test sensitivity. Forensic testing programs typically implement procedures to minimize tampering, and current federal protocols monitor creatinine, temperature, pH, specific gravity, and oxidizing adulterants.^6^

Because marijuana use is common, tetrahydrocannabinol (THC) is a frequently screened substance. The lateral flow immunoassay (LFI), depicted in Figure 1A, provides rapid, low-cost detection of THC but is vulnerable to interference, particularly from sample matrix effects or adulteration.^7–10^ The primary urinary metabolite, 11-nor-9-carboxy-tetrahydrocannabinol (THC–COOH), a nonpsychoactive compound excreted after cannabis consumption (Figure 1B),^11^ is semiquantitatively detected when the analyte is above the standard cutoff for THC of 50 ppb.^3^ When an initial LFI result is negative (i.e., below the cutoff), confirmatory analysis using mass spectrometric methods such as gas chromatography–mass spectrometry (GC–MS) is typically not performed, and the individual is considered to have passed screening. Thus, individuals seeking to conceal drug use have developed methods to adulterate urine samples in order to produce a false negative result.

**Figure 1:**
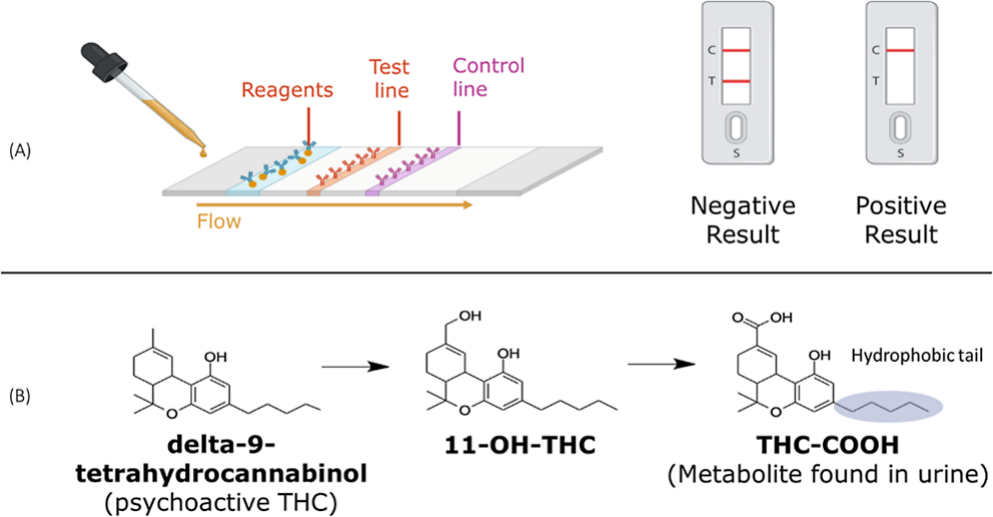
Details of lateral flow immunoassay tests based on competitive antibody binding. (A) Schematic for detection THC in urine and (B) the detected metabolite, THC–COOH, when above 50 ppb is a typical positive.

One method of adulteration is the use of benzalkonium chloride (BAK), a quaternary ammonium compound widely used as a preservative and antimicrobial agent in disinfectants, ophthalmic formulations, and topical antiseptics, which has emerged as an adulterant capable of producing false-negative results.^12^ BAK is actually a mixture of alkylbenzyldimethylammonium chlorides with the general chemical formula C_6_H_5_CH_2_N­(CH_3_)_2_RCl, where R denotes long, hydrophobic alkyl chains typically containing 12 to 18 carbon atoms (Figure 2A). The surfactant properties of BAK are attributed to its amphiphilic structure that features a quaternary ammonium polar headgroup that is hydrophilic and a long alkyl chain that is hydrophobic allowing it to orient at interfaces and self-assemble into micelles. Reports of BAK adulteration have been observed across multiple immunoassay platforms, including solution-based automated analyzer systems such as EMIT (enzyme multiplied immunoassay technique) and TDx (fluorescence polarization immunoassay) as well as point-of-care tests such as lateral flow immunoassays (LFIs). In commercial consumer products, BAK is typically used at concentrations ranging from 0.004 to 0.13% w/v (40–1300 ppm).^13^ The previously proposed mechanism by which BAK masks the presence of THC–COOH involves micelle formation,^12^ which consists of nanoscale aggregates of surfactant molecules that interact with THC–COOH and disrupt antibody–antigen interactions within LFI test lines (Figure 2B). Such interference may lead to false-negative results, compromising the reliability of the immunoassay-based screening programs. Despite this vulnerability, BAK is not included in standard adulterant panels and is challenging to detect rapidly.^14^ A smartphone-based bromothymol blue method has also been reported,^15^ offering a portable approach for analysis; however, it requires specialized software and may exhibit nonspecific interactions between the dye and urine matrix constituent.

**Figure 2:**
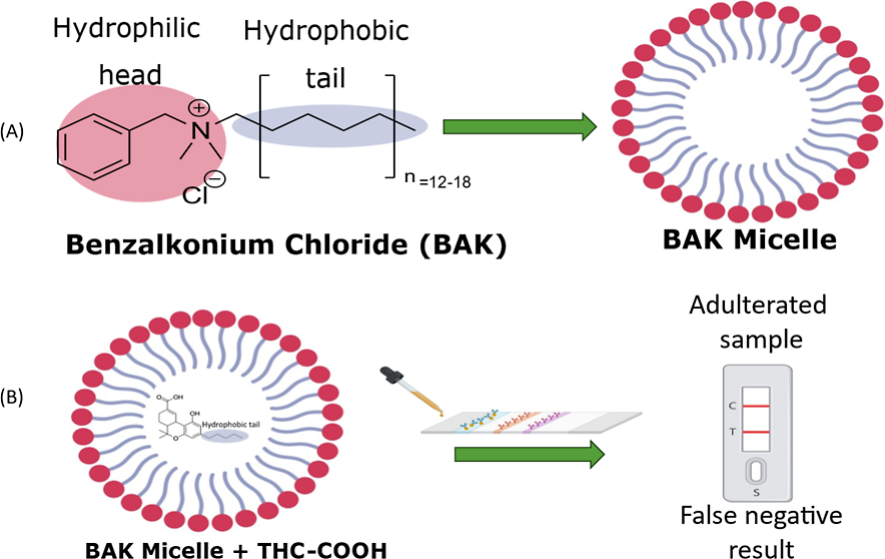
Benzalkonium chloride (A) structure and micelle formation and (B) proposed mechanism of adulteration of THC LFI tests due to THC–COOH incorporated into the micelle.

In our study presented herein, we address these issues and potential limitations by (i) evaluating the impact of BAK concentrations typical of consumer products on THC–COOH detection in LFIs and by (ii) developing an instrumentation-free approach to identify adulterated samples. Our method implements the standard addition method to distinguish true negatives from BAK-induced false negatives, offering a practical strategy for strengthening routine THC urine screening.^16^

## 2 Experimental Methods

### 2.1 Materials and Reagents

Two different LFI test kits for measuring THC use in urine were used for this study. The iCassette single THC test device, 1-DTH-102, (Abbott Toxicology, Portsmouth, VA, USA) and Easy Home Single Screen Cannabinoid Drug Test, EDTH-114, (Burr Ridge IL, USA) had previously been assessed, had a positive threshold of 50 ppb of THC–COOH, and had excellent sensitivity and specificity.^17^ These kits are denoted as iCas and EZH in the data tables. The concentration of positive urine samples was determined using a U-Catch marijuana multilevel drug test card designed to detect 20–300 ppb (Shenzhen Bioeasy Biotechnology Co., Shenzhen PRC). Negative and positive urine samples were collected from healthy volunteers. Additional confirmed-positive THC urine samples were created by volumetric addition of THC–COOH:(−)­11-nor-9-carboxy-delta 9-THC, 1 mg/mL in methanol (T-019, Supelco-Cerilliant Corp., Round Rock, TX, USA), yielding final THC–COOH concentrations ranging from 100 to 400 ppb. Negative control urine, S-020, was purchased from Cerilliant Corporation (Round Rock, TX, USA).

Specimen validity testing before and following the addition of BAK was accomplished with i-Screen Specimen Validity Test strips, I-DUC-111 (Abbott Toxicology, Pomona, CA, USA). Two BAK stock solutions were used. One BAK stock was an aqueous solution in distilled water from a BAK solid, B6295 (Sigma-Aldrich, St. Louis, MO, USA). The second BAK stock was purchased as a standard solution at 50% w/w, BAK B20760 AP (Thermo Scientific, Lancashire, UK). These stock solutions were used for adulteration and standard calibration curves to determine the BAK concentration in consumer products.

Other reagents included Eosin Y, BA1610-772016 (J.T. Baker, Phillipsburg, NJ, USA), used to assess the BAK concentration in consumer products made as a stock solution of 5 × 10^–4^ M Eosin Y in water, and assays were conducted at a concentration of 1.7 x10^–6^ M. Various consumer products containing BAK were purchased from a local grocery store (Seattle, WA).

### 2.2 Instruments

Visible spectra over a wavelength range of 380–780 nm were recorded on a Vernier, VSP-UV spectrophotometer (Beaverton, OR, USA). Photographs of LFI and specimen validity results were acquired using iPhone cameras.

### 2.3 Procedure for Identification of Urine Samples Adulterated with BAK

All urine samples that tested negative for THC (including true negative and false negatives due to BAK masking) were subject to secondary testing. For this step, 800 μL of urine was added to a 2 mL polypropylene test tube with 200 μL of 500 ppb THC–COOH standard, well mixed, and tested again with a new LFI test kit. These resulting specimens contained a minimum THC–COOH concentration of 100 ng/mL and, therefore, should produce a positive result on an LFI test with a 50 ppb THC–COOH cutoff unless adulteration caused false-negative interference.

## 3 Results and Discussion

### 3.1 Evaluation of BAK Concentrations in Consumer Product and the Association of Masking with the Critical Micelle Concentration

A central question in evaluating BAK-induced interference is whether false negatives arise from micelle formation, which could entrap THC–COOH and prevent its binding to antibodies affixed to the test line of the LFI apparatus. If micelle entrapment is a plausible underlying mechanism, masking should occur once BAK concentrations exceed the critical micelle concentration (CMC). Salient information for this study is provided in Table 1. Reported CMC values for BAK, listed in Table 1(A), range from 50 to 500 ppm after conversion to consistent concentration units.^18–20^ To determine whether these concentration levels are reached by common commercial products used for adulteration, and because benzalkonium chloride (BAK) levels are not publicly disclosed for many formulations, an assay was developed to estimate BAK content in commercial products. To explore the relationship of masking with BAK concentration, ten commercial products (many shown in Figure 3) were significantly diluted and analyzed with a dye-binding assay for detection of cationic surfactants using Eosin Y.^21^ The spectral shift of Eosin Y in the presence of BAK is shown in Figure 4 with an inset calibration plot prepared from replicate analyses of liquid BAK stock standards.

**Table 1:** BAK CMC and Consumer Product Concentration Data^a^

A. literature values for CMC of BAK in various matrices			
matrix	reported CMC (ppm, w/v)	reference	
water	500	^18^	
2.4% w/v NaOCl	80	^19^	
50 mM NaCl	50	^20^	
B. concentration of BAK from product label or measured from Eosin Y calibration			
ID	product (brand/type)	[BAK] from Label (ppm, w/v)	[BAK] measured (ppm, w/v)
101	Bausch and Lomb/Eye Wash	100	100
102	Band-Aid/Antiseptic Liquid	1300	2000
103	Visine Advanced/Eye Drops	120	100
104	Clear Eyes/Eye Drops	NR	140
105	Burt’s Bees/Dog Eye Wash	NR	121
106	Signature Care/Dry Eye Drops	NR	110
107	Walgreens/Eye Drops	NR	130
108	Visine Total Comfort/Eye Drops	NR	71
109	Pure Aid/Eye Drops	NR	29
110	Burt’s Bees/Eye Wash	NR	130
111	Simple/Micellar Water	0^b^	168^c^

a NR = not reported on the label or in product literature.

b Product does not contain BAK; manufacturer-reported formulation contains cetrimonium chloride and cetylpyridinium chloride.

c Effective BAK concentration projected from a nonspecific Eosin Y signal resulting from interaction with cationic surfactants; signal is not BAK-selective.

**Figure 3:**
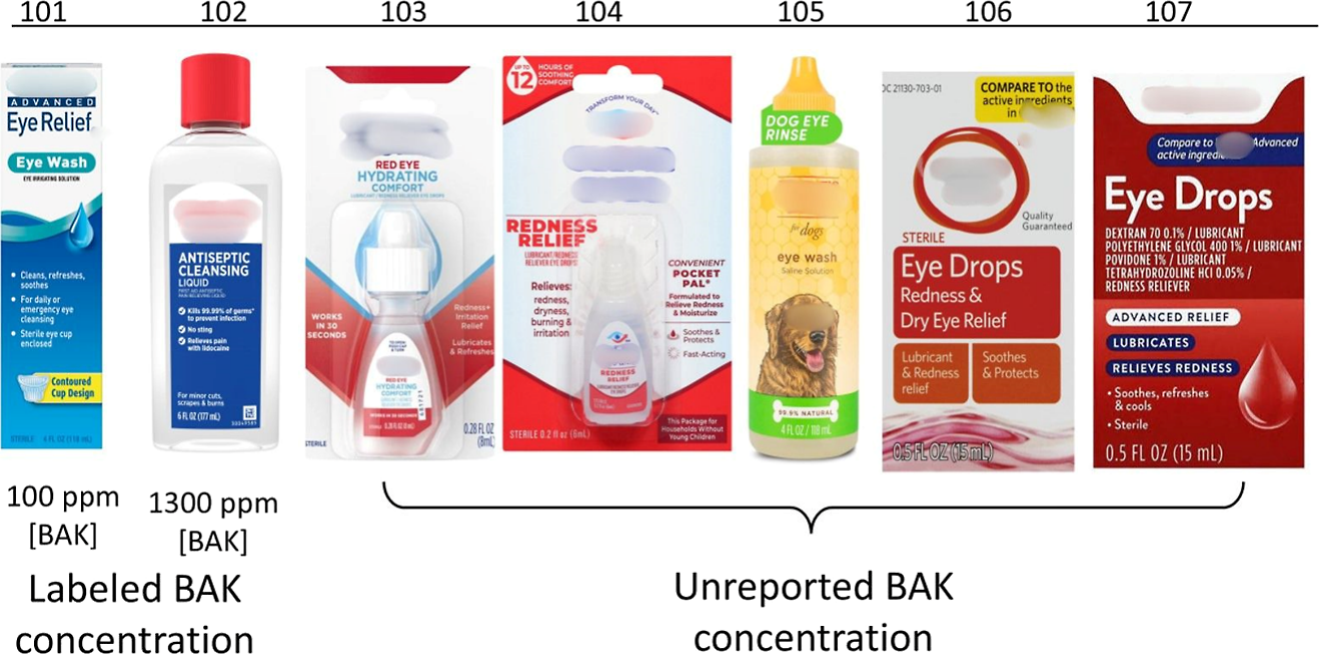
Representative commercial products tested for BAK concentration and efficacy as adulterants in THC testing.

**Figure 4:**
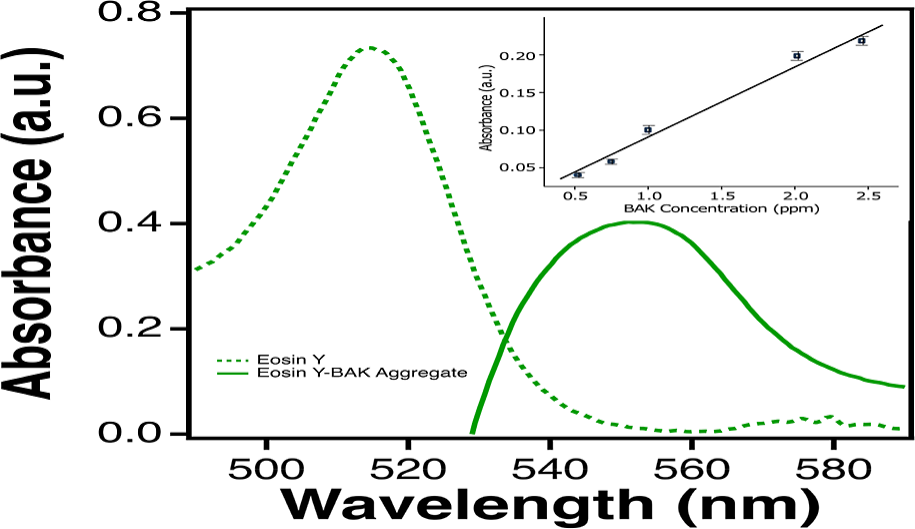
Spectral data for quantitative measurement of BAK using Eosin Y-binding UV–vis spectrophotometry. Linear regression statistics of the inset calibration plot at 556 nm are *y* = 0.0929*x* – 0.0017, *R* ^2^ = 0.981.

Quantitative analysis of benzalkonium chloride (BAK) in commercial products, summarized in Table 1(B), yielded concentrations ranging from 29 to 2000 ppm. Two surfactant-based ophthalmic products with labeled BAK concentrations (#102 and #103) either matched the stated values or were within 20% of their label claims, supporting the overall accuracy of the assay in matrix-matched formulations. For the remaining commercial eye care products (#104–1101), the reliability of the measured BAK concentrations is supported by literature reports indicating typical BAK levels of approximately 100 ppm in ophthalmic formulations,^13^ which are consistent with values obtained using the dye-binding assay. The antiseptic liquid (#101), which has a publicly reported BAK concentration, showed a measured value slightly higher than the labeled concentration (2000 ppm vs 1300 ppm). Because extensive linearity and precision validation were not performed across this concentration range, the labeled value for #101 was used for subsequent calculations involving this product. Additionally, product #111, a micellar water containing the cationic surfactants cetrimonium chloride and cetylpyridinium chloride rather than BAK, also produced a measurable response in the Eosin Y dye-binding assay, yielding an estimated BAK-equivalent concentration of 168 ppm. This finding demonstrates limited specificity of the assay for BAK, confirming that other cationic surfactants can contribute to the observed absorbance response. This product was included in the following adulteration studies to preliminarily evaluate the masking potential of alternative consumer surfactants, either as the sole surfactant in the formulation or in combination with BAK.

The quantitative data in Table 1(B) describing concentrations in commercial products were used to assess the effect of BAK on urinary THC testing by systematically varying the concentration through controlled volume additions to a series of positive THC–COOH urine samples. Figure 5 presents LFI results for a representative sample with a THC–COOH concentration of 100 ppb, tested across increasing BAK concentrations. At concentrations below 200 ppm, LFI results remained positive, indicating that BAK levels were insufficient to cause interference and produce a false negative. At concentrations greater than 400 ppm, the results turned negative. Using concurrent dilution studies, it was confirmed that the observed results attributable to commercial product addition were not due to dilution reducing THC–COOH concentrations below the detection threshold but rather were indicative of BAK acting as an effective adulterant.

**Figure 5:**
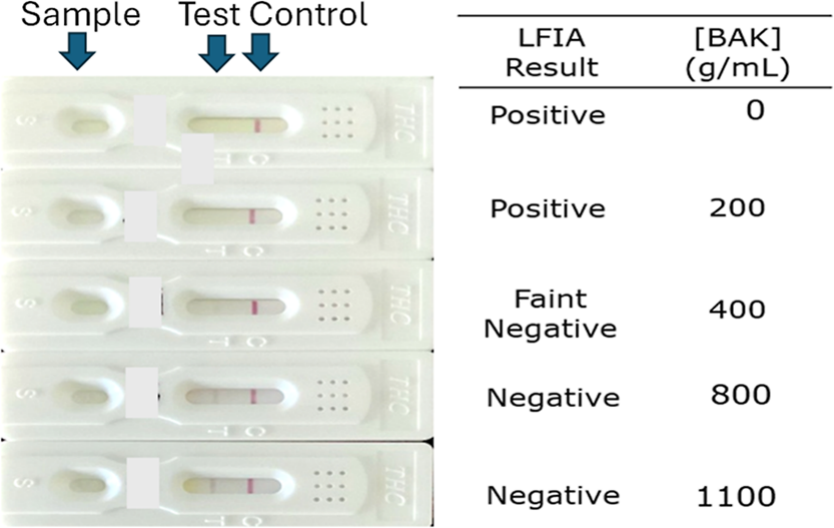
Titration of BAK aqueous standard adulteration in urine Matric ID-A containing 100 ppb THC–COOH.

To determine whether BAK-adulterated samples would be flagged by standard drug-testing protocols, additional analyses were performed on samples using the specified adulteration test strips that measure six metrics used to detect adulteration: creatinine, nitrites, glutamine, pH, specific gravity, and oxidants. Representative results from two samples across varying BAK concentrations are shown in Figure 6. Despite elevated BAK concentrations, adulteration-induced false-negatives remained undetected, preventing the identification of tampered samples. These findings align with prior reports indicating that BAK adulteration can evade standard specimen validity tests^14^ and suggest that adulteration occurs when BAK is present at concentrations near the literature-reported CMC values, as shown in Table 1. This supports the hypothesis that micelle entrapment of THC–COOH, rather than dilution or a degradative chemical process, underlies the observed false-negative results.

**Figure 6:**
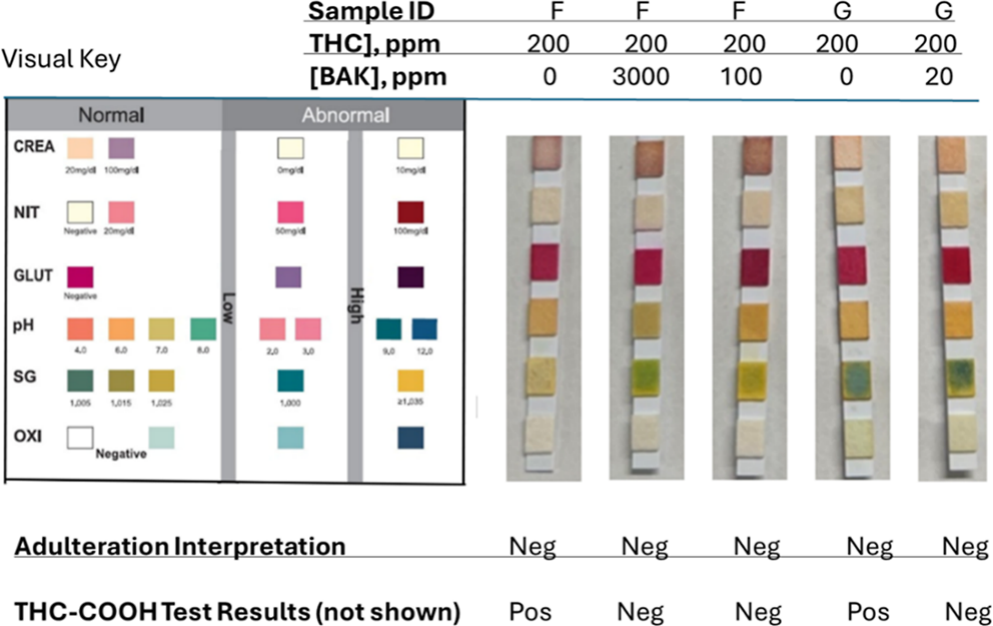
Example specimen validity testing results for two positive urine samples each containing 200 ppb THC–COOH. Sample F was spiked with 3000 ppm BAK stock and 100 ppm commercial antiseptic liquid (Product #102). Sample G was spiked with name brand eye drops (Product #103) containing BAK.

## 3.2 Critical Micelle Concentration and Estimated Volumes of BAK-Containing Products Required for Adulteration

Adulteration data from seven positive urine samples with varying THC–COOH concentrations above the positive testing threshold (range 100–600 ppb) are summarized in Table 2. To model real-world adulteration scenarios, various volumes and resulting concentrations of BAK were added to samples to approach the conditions that induce false negatives. The BAK concentrations causing interference varied by sample, ranging from ∼20 to 1000 ppm, values that are in alignment with the BAK concentration range of reported CMC values in Table 1A.

**Table 2:** Representative Masking Data for Various BAK Solutions with Seven Urine Samples^a^

urine ID	[THC–COOH] (ppb, w/v)	product ID	[BAK] (ppm, w/v)	LFI result	masking observed	estimated CMC (ppm, w/v)
A	100	BAK stock	200^b^, 400^b^	+, −	no, yes	<400
B	200	BAK stock	700^b^, 800^b^	+, −	no, yes	<800
B	400	BAK stock	800^b^, 1000^b^	+, −	no, yes	<1000
B	600	BAK stock	800^b^, 1000^b^	+, −	no, yes	<1000
C	150	antiseptic #102	433^b^, 650^c^	–	yes, yes	<433
C	150	eye drop #103	40^c^, 60^d^	–	no, yes	<60
C	150	eye drop #104	33^c^, 50^d^	+, −	no, yes	<50
C	150	dog eye wash #105	47^c^, 70^d^	+, −	no, yes	<70
C	150	eye drop #107	40^c^, 60^d^	+, −	no, yes	<60
C	150	eye drop #108	43^c^, 65^d^	+, −	no, yes	<65
C	150	eye drop #109	24^c^, 36^d^	+, −	no, yes	<36
C	150	eye wash #110	10^c^, 15^d^	+, −	no, yes	<15
C	150	micellar H_2_O #111	0^c^ ^,^ ^e^, 0^d^ ^,^ ^e^	–, –	yes, yes	not BAK
D	160	BAK stock	50^b^, 100^b^	+, –	no, yes	<100
E	150	BAK stock	50^b^, 100^b^	+, –	no, yes	<100
F	200	antiseptic #102	100^b^	–	yes	<100
G	200	eye drop #103	20^b^	–	yes	<20

a The urine volume:commercial product volume ratio.

b >3:1.

c =2:1 and.

d =1:1.

e No detectable BAK was observed.

Variability between observed masking concentrations and published CMC values are expected, as several factors influence this relationship. First, BAK is a mixture of surfactants with varying hydrophobic tail lengths (typically 12–18 carbons in the alkyl chain), and its exact composition depends on the formulation source, which can affect CMC. Several of the literature CMC values were converted from mol/L to ppm requiring an estimate of the average molecular weight of BAK. Herein, an average BAK molecular weight of 339 g/mol was assumed, corresponding to the selection of the mean hydrophobic tail length of 15 carbon atoms. Second, the CMC of BAK is influenced by ionic strength because of its positively charged headgroup. Increasing ionic strength promotes micelle formation, thereby lowering the CMC.^22^ Human urine typically has high ionic strength, ranging from 200 to 600 mM for a 24 h collection.^23,24^ Consequently, the CMC is matrix-dependent and may vary across individual urine samples. For this study, the most relevant CMC literature value is ∼50 ppm in 500 mM NaCl.^20^ A third factor limiting correlation of the occurrence of masking (i.e., false negative results) at a given BAK concentration with published CMC values is that commercial products containing BAK can contain other surface-active agents and exhibit high variability in ionic composition. Estimating the CMC based solely on BAK masking concentration introduces variability, as unknown matrix effects and formulation differences impact the results.

Another remaining question in evaluating the relationship among BAK concentration, the CMC, and effective adulteration is the mechanism by which THC–COOH becomes associated with micelles. One possibility is the formation of mixed micelles, in which the hydrophobic moiety of THC–COOH partitions into the BAK micelle, thereby contributing to micelle formation and influencing its apparent CMC in solution.

To examine the effects of BAK independent of the sample matrix, urine samples (ID-A and ID-B, Table 2) were spiked with high-concentration BAK stock solutions. This approach minimized the final volume required for effective adulteration and excluded interference with other commercial ingredients. For these samples, BAK stock solutions were added such that the urine-to-BAK volume ratio remained greater than 3:1. Although the data set is limited, the observed trends suggest that the CMC associated with THC–COOH masking occurs at higher BAK concentrations than those found in commercial products containing BAK. Additionally, BAK stock additions to the two urine samples (ID-A and ID-B) suggest a possible correlation between the THC–COOH concentration and the apparent CMC. The implications of these findings for the mechanism of BAK interference with THC–COOH detection in LFI analysis remain unclear, as they may be influenced by the variability among the urine samples tested or the semiquantitative nature of the LFI testing.

While BAK stock solution additions helped to demonstrate the BAK concentration-dependent nature of masking in a controlled laboratory setting, it does not fully simulate real-world adulteration using commercial products, most of which likely contain BAK at lower concentrations. To produce a false-negative urine test result through adulteration, benzalkonium chloride (BAK) must be added to the sample immediately after collection and maintained near body temperature, as analyte concentrations, adulterants, and sample temperature are rapidly evaluated following collection. However, if an excessive amount of a commercial product containing BAK is added, then the sample may fail validity testing due to dilution. To determine the volume required to produce a negative THC result, nine different commercial products containing BAK were added to the same urine sample (ID-C) containing 150 ppb THC–COOH. Because of the lower concentration of the commercial product compared to BAK stock, two additional urine-to-product volume ratios of 2:1 and 1:1 were tested, as indicated in Table 2. After adulteration, the THC–COOH concentration was either 75 or 100 ppb, levels well above the positive test threshold in the absence of matrix interference with BAK. This study revealed that two out of nine samples masked THC when added at a urine-to-commercial product ratio of 2:1 The first, product #102, a highly concentrated antiseptic liquid, was expected to mask, as this product has a labeled concentration of 1300 ppm BAK and is expected to be above the CMC at this ratio. The second case of interference at a urine-to-commercial product ratio of 1:1 involved micellar water (product no. 111). This result was not necessarily expected as BAK was not listed as an ingredient on the product label. However, the label did indicate the presence of two cationic surfactants, cetrimonium chloride and cetylpyridinium chloride, suggesting similar surface-active properties to BAK that could account for the observed masking effect. The dye-binding assay estimated a BAK concentration of 168 ppm, likely reflecting matrix susceptibility and signal produced by other surfactants, with the limited specificity of the eosin Y assay.

In the remaining 7 of 9 samples (all either eyewash or eyedrop products), masking was observed at a urine-to-product volume ratio of 1:1. These products had measured BAK concentrations ranging from 29 to 140 ppm, resulting in final BAK masking concentrations of approximately 15–70 ppm after dilution. These concentrations generally fall within the reported CMC range for BAK in high ionic strength solution, supporting micellization as an adulteration mechanism. In a previous study to identify adulteration by eyedrops, all samples were tested at a urine-to-commercial product ratio of 0.67 (10 mL urine and 15 mL eyedrops), supporting the current finding that a ratio of ∼1:1 is generally required to produce masking with typical eyedrops.^14^ The corresponding conclusion is that an equal value of eyedrops and urine would be needed to mask THC usage. For example, a typical urine sample of ∼50 mL would require a similar volume of eyedrops with a ∼100 ppm BAK concentration. Less product can be used if it contains a higher concentration of BAK such as an antiseptic liquid. The volume of a commercial product required to produce masking has important implications, as such, dilution may be detected by programs that perform specimen validity testing. Dilution could reduce validity markers such as creatinine concentration or specific gravity below acceptable thresholds, leading to samples being flagged for potential adulteration. However, dilution or adulterant was not observed in the samples analyzed in this study and would vary between samples and testing programs, particularly depending on whether specimen validity or adulteration checks are implemented. Although routine chemical tests did not detect dilution or BAK adulteration, the addition of a large volume of a room-temperature solution could potentially lower specimen temperature below the acceptable range used in forensic and clinical testing. Because samples found to be below body temperature immediately after collection are considered invalid, specimen temperature monitoring may help identify adulterated samples when the commercial product has not been warmed to body temperature prior to addition to urine.

### 4.1 Development and Evaluation of a Method to Detect BAK Adulteration in Urine Samples

Adding BAK to urine constitutes a stealthy and effective form of adulteration. In this context, BAK functions as a negative-dependent matrix interferer that suppresses the analytical detection of THC–COOH. A classic strategy for addressing dependent interferences in quantitative analysis is the standard addition method (SAM).^16^ In the context of our study, applying the SAM involves measuring the analyte (THC–COOH) in an aliquot of its native matrix and then spiking an initially equivalent aliquot of the sample with a known concentration of THC–COOH standard and comparing the signals to assess interference. The SAM provides excellent analytical accuracy because the added standard is subject to the same matrix interference as the original analyte. Although lateral flow immunoassays are only semiquantitative, we hypothesized that SAM could effectively detect BAK adulteration as a matrix interference.

As conceived, applying the SAM involves testing all negative THC–COOH results with a second LFI test following the scheme depicted in Figure 7. A small volume of each urine sample that tested negative is removed via a pipet and transferred to a tube containing a standard concentration of THC–COOH. After mixing and resting for 2 min, this dilution is tested with a new LFI test. A negative second test signals the presence of a masking substance such as BAK and indicates that the initial result is likely to be a false negative.

**Figure 7:**
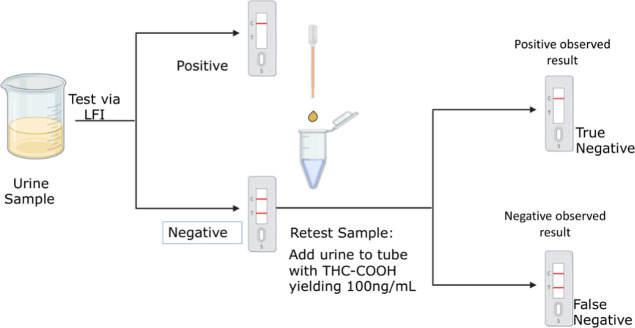
Standard Addition Method applied to identifying potential false negatives caused by BAK adulteration.

To evaluate the sensitivity of applying SAM to detect BAK adulteration, 45 urine samples were tested using two different LFI kits (iCass and EZH). The sample set included 15 true positives, 15 true negatives, and 15 BAK-induced false negatives. True positive urine samples contained between 100 and 400 ppb of THC–COOH. False negatives were generated by adding 800 ppm BAK standard to positive samples and confirming masking with a negative LFI result. All samples passed standard adulteration screening and then were used in a blind study conducted in an upper-division forensic science laboratory where students were unaware of each sample’s identity. Working in pairs, students classified each sample as positive or negative based on the SAM procedure in Figure 7. To identify BAK adulteration, all samples that tested negative in round 1 were further evaluated. For round 2 testing based on SAM, 800 μL of each negative sample was transferred to a tube containing 200 μL of 500 ppb THC–COOH standard, resulting in a final concentration of 100 ppb—twice the LFI cutoff. If the second test is positive, the added THC–COOH is detected, indicating that BAK is unlikely to be present at levels that would mask the drug test result and can be considered a true negative result. In contrast, if the second test remains negative, this may indicate the presence of an adulterant such as BAK, as the results suggest that something in the sample is interfering with detection of the added THC–COOH, potentially through micellar entrapment that limits LFI detection and is deemed a false negative result.

Results from the 45 blind samples are summarized in Table 3. In round 1, the presence of 15 false negatives, caused by BAK interference, reduced sensitivity, yielding a true positive rate of 50%. Round 2 of testing applied the SAM procedure to all 30 samples that initially tested negative. Results were evaluated based on whether they aligned with the sample’s true identity, specifically whether spiked THC–COOH was detected. All 15 true negatives spiked with THC–COOH tested positive, while all 15 false negatives remained negative, confirming BAK interference. When results from both rounds of testing were combined, no false results were observed or remained for the two different LFI products used in testing, demonstrating that the SAM method identified BAK-adulterated samples with 100% sensitivity and specificity. This blind study demonstrates the potential of the SAM in detecting BAK-adulterated samples. SAM could be incorporated into drug testing kits as a routine adulteration check. A proposed kit would include: a urine collection cup, two LFI devices, a graduated plastic tube containing a small volume of THC–COOH standard, and a disposable pipet for transferring urine.

**Table 3:** Results of Blind Testing of 45 Urine Samples for THC–COOH as Submitted (Round 1 Test) and after Employing a Method for Identifying Adulteration with BAK (Round 2 Test)

A. blind test results				
sample ID	test name	results samples test 1	results samples test 2 + spike	spike test interpretation
true positives (TPs)	iCas	15/15 (+)	NA	NA
	EZH	15/15 (+)	NA	NA
true negative (TN)	iCas	15/15 (+)	15/15 (+)	confirms these samples are TN
	EZH	15/15 (+)	15/15 (+)	confirms these samples are TN
false negative (FN)	iCas	15/15 (−)	15/15 (−)	confirms these samples are FN
	EZH	15/15 (−)	15/15 (−)	confirms these samples are FN
B. testing validity calculations^a^				
specimen validity equation	calculated results test 1	calculated results test 2 + spike		
specificity = TN/(TN + FP)	100%	100%		
sensitivity = TP/ (TP + FN)	50%	100%		

a No false positives (FPs) in the sample set.

Practical considerations for implementing this proposed unmasking assay include the additional cost of a second LFI, which is typically less than one dollar for a THC-only test. Another concern is the potential for accidental spillage or contamination of a negative urine sample with the THC–COOH standard, creating a false positive in round 1 of screening. Although the small volume and concentration of the standard are unlikely to cause a false positive in a full-volume negative urine sample, this risk could be mitigated by sealing the tube with a tamper-evident seal, to be broken only if the sample yields a negative test result, thereby preserving the integrity of round 1 of testing. Another consideration is the stability of THC–COOH in the SAM testing tube during storage and transport. This is important, as BAK detection depends on THC–COOH remaining intact and detectable by the LFI.^25^ This concern is especially relevant for nonlaboratory settings, such as home drug testing, where storage conditions may vary. Adopters of the SAM method to identify adulteration could address this concern through stability testing or protective packaging that limits metabolite degradation under typical home storage conditions. While the use of the SAM to identify the likely presence of BAK does not confirm THC–COOH presence in the original sample, it flags potential adulteration and indicates the need for recollection under supervised conditions or confirmatory testing. Previous studies have demonstrated that GC–MS methods are not impacted by BAK interference, as chromatographic methods eliminate surfactant-related matrix effects.^7,12^

## 5 Conclusions

This study demonstrates that solutions containing BAK can effectively adulterate urine samples and interfere with the detection of THC–COOH in lateral flow immunoassays, leading to false-negative results. Adulteration occurred at BAK concentrations consistent with or near its critical micelle concentration (CMC), supporting a micelle-mediated masking mechanism. The variability in masking thresholds across urine samples reflects the influence of matrix composition and highlights the unreliability of estimating the CMC solely from product labels or concentrations. A dye-binding assay using eosin Y enabled estimation of BAK concentrations in various commercial products, revealing sufficient levels to mask THC under realistic tampering scenarios without being detected by typical adulteration screening.

An analytical method using the SAM was developed and validated as a reliable approach for detecting BAK adulterated samples to enhance the integrity of urine drug testing. In a blinded undergraduate study, the SAM approach achieved 100% sensitivity in identifying false negatives caused by BAK. This method, which involves rerecording negative samples after spiking with THC–COOH, can be easily incorporated into routine drug testing protocols with minimal cost and operational burden. While SAM does not confirm the presence of THC–COOH in the original specimen, it flags samples for recollection or confirmatory testing by GC–MS, a method that is not subject to BAK adulteration. The incorporation of this new method for BAK adulteration identification enhances the robustness of urine drug testing, particularly in unsupervised or at-home settings, and offers a practical countermeasure against a deleterious form of adulteration.
